# *In vitro* rumen fermentation and protein protection of canola and soybean meals using tannin extract from *Bauhinia hookeri*

**DOI:** 10.1093/tas/txaf144

**Published:** 2025-11-04

**Authors:** Bereket Zeleke Tunkala, Kristy DiGiacomo, Pablo Alvarez Hess, Frank Dunshea, Brian Leury

**Affiliations:** Faculty of Science, The University of Melbourne, Parkville, VIC, 3010, Australia; Faculty of Science, The University of Melbourne, Parkville, VIC, 3010, Australia; Agriculture Victoria Research, 1301 Hazeldean Road, Ellinbank, VIC, 3821, Australia; Faculty of Science, The University of Melbourne, Parkville, VIC, 3010, Australia; Faculty of Biological Sciences, The University of Leeds, Leeds, LS2, 9JT, United Kingdom; Faculty of Science, The University of Melbourne, Parkville, VIC, 3010, Australia

**Keywords:** ammonia-N, condensed tannin, feed additives, gas production, methane, protein fractions, rumen fermentation, ruminants

## Abstract

Tannins are natural compounds known to suppress methane-producing microbes and bind dietary proteins in the rumen, potentially improving nitrogen use efficiency. This study evaluated the effects of condensed tannin extract (TE) from *Bauhinia hookeri* hay on in vitro rumen fermentation and protein degradation of canola and soybean meals. We hypothesised that TE would suppress methane production and enhance protein protection in both meals. Each meal was treated with 0%, 2%, 4%, or 6% TE in dry matter basis and incubated for 24 h using the ANKOM gas production system. Total gas production was reduced in treated canola meal by 16.4% at 2% TE (*P* = 0.014), 30.1% at 4% TE (*P* < 0.001), and 52.7% at 6% TE (*P* < 0.001). Methane production was unaffected at 2% TE (*P* = 0.267) but declined by 37.2% at 4% TE and 40.4% at 6% TE (*P* < 0.001). Lag time before gas production began increased in both feeds (*P* < 0.05). Total volatile fatty acid (VFA) concentration of soybean meal was unchanged at 2% and 4% TE but declined at 6% TE (*P* = 0.006). Soluble protein (fraction ‘a’) decreased in both meals with TE inclusion (*P* < 0.001), while the slowly degradable protein fraction (‘b’) increased (*P* < 0.05). The degradation rate of fraction ‘b’ was reduced across all TE-treated groups (*P* < 0.05). These results suggest that TE from *Bauhinia hookeri* hay can reduce methane emissions and protect protein from excessive ruminal degradation. The 4% inclusion rate consistently showed optimal results across fermentation and protein parameters, making it a promising level for practical application.

## Teaser

This study highlights the potential of Bauhinia hookeri tannin extract as a natural feed additive to reduce methane emissions and protect dietary protein in ruminants. A 4% inclusion level was most effective, offering a sustainable strategy to enhance livestock productivity and environmental outcomes.

## Introduction

Excreted nitrogen in the faeces and urine of ruminants can account for up to 70% of ingested nitrogen, especially when the animal is consuming rapidly degradable protein (Huws et al 2018; Li et al 2020). Nitrogen loss could result in increased environmental pollution as urea, nitrous oxide, and ammonia, and economic damage through declining production performance (Dijkstra et al 2013; Yáñez-Ruiz et al 2017; Yang et al 2021). The biological value of protein is also depleted when rumen microorganisms ferment excessive degradable protein (Garg 1998). Therefore, protection of crude protein (CP) is crucial to reduce extensive rumen degradation and excretion, and helps to increase available bypass protein in ruminants (Boucher et al 2009). Furthermore, post-ruminal utilisation of protein eliminates liver stress caused by excessive ammonia concentrations and energy losses associated with protein degradation in the rumen (Kamalak et al 2005).

Considerable variation is reported among feeds in N efficiency, indicating an opportunity to manipulate feeds for better N use (Dijkstra et al 2013). Untreated soybean and canola meals are good sources of digestible protein but ineffective in providing rumen undegradable protein attributed to their extensive degradation in the rumen (Wright et al 2005; El-Waziry et al 2007). Therefore, several studies have been undertaken to identify potential protein protection options.

Protein protection is possible by using feed additives and chemicals, heating, pelleting, feeding protected amino acids, use of plant secondary metabolites, and inhibition of microbial proteolytic activity (Kamalak et al 2005; Lee et al 2015). However, the incorporation of chemicals and antibiotics as feed additives in the diet of livestock is less desirable for the possible contamination of animal products (Barton 2000) and the development of chemical resistance. Although widely used to increase rumen undegraded protein and reduce antinutritional factors, extended heating can denature essential amino acids (Kung and Rode 1996), create Maillard reactions between amino acids and sugars (Broderick et al 1991), and peptide bonds between different amino acids, resulting in resistance to enzymatic breakdown (Kamalak et al 2005). Feeding separately protected amino acids do not protect the protein in the feed but provides additional amino acids not available for rumen degradation. Therefore, using protected amino acids does not reduce the excessive production of ammonia, loss of nitrogen and conveys additional cost (Kaufmann and Lüpping 1982). For this reason, attention has shifted towards natural supplementation as a safe means of modifying ruminal fermentation including protein protection for the efficient use of protein concentrates in ruminants (Naseri et al 2013).

Condensed tannins have a strong ability to bind with CP, producing a tannin-protein complex formed by bonding between the phenolic groups of tannin and the carbonyl groups of proteins (Getachew et al 2000; Mueller‐Harvey 2006). The bonding reduces the availability of substrate for bacterial colonisation and ruminal fermentation, thereby decreasing the rumen degradability of CP, improving protein availability in the intestine and enhancing animal production efficiency (Jackson et al 1996; Fondevila et al 2002; Grainger et al 2009). However, the effect of condensed tannin depends on its dose, source, bioactive molecule composition, structure of tannin and feed protein characteristics (Mueller‐Harvey 2006; Huang et al 2018). Therefore, studies on the evaluation of different tannin sources and optimum inclusion levels in ruminant diets are essential.

*Bauhinia hookeri* is a native Australian leguminous plant, grows predominantly in North East Queensland, and is rich in total phenolic (160 mg/g) and condensed tannin contents (67.4 mg/g) (Tunkala et al 2023c). The phenolic composition is also diverse. This concentration and diversity could be used as a potential natural alternative for different purposes. To the best of our knowledge, no report is available on using *Bauhinia hookeri* hay as a source of tannin for methane reduction and protein protection in ruminants. This research was conducted to determine the effect of the TE from *Bauhinia hookeri* hay on *in vitro* fermentation characteristics and evaluate the effect of three TE inclusion rates on methane proportion in headspace gas and protein protection of protein-rich feeds. It was hypothesised that mixing limited amounts of tannin extract (TE) from *Bauhinia hookeri* hay with canola meal or soybean meal would increase the proportion of rumen undegradable protein, reduce the amount of ammonia-N released, methane percentage and *in vitro* degradable protein.

## Material and methods

All procedures were conducted per the Australian Code of Practice for the Care and Use of Animals for Scientific Purposes (NHaMRC 2013). The Department of Jobs, Precincts and Regions Agricultural Research and Extension Animal Ethics Committee approved the preparation and use of cannulated cows from which rumen fluid was sourced for this experiment.

### Substrates, substrate preparation and treatments

Canola meal (38.0% CP) and soybean meal (49.6% CP) were used as substrates because they are commonly used protein supplements for ruminants. Both substrates were solvent-extracted and purchased from an Australian commercial supplier (Peter Gibbs Stock Feeds, Australia). The feed samples were ground into 2 mm particle diameter using grinder (Breville, The Coffee & Spice Grinder, Stainless Brushed Steel, Myer) and sieved to ensure the size. The leaves of *Bauhinia hookeri* were collected from Townsville, Queensland then sun-dried to hay. *Bauhinia hookeri* was selected as a tannin source for its high phenolic and condensed tannin contents.

The TE of *Bauhinia hookeri* was extracted with a minor modification to the protocol described by Medini et al (2014) using methanol. The *Bauhinia hookeri* hay was ground in to 2 mm particle size, mixed with 4 mL/g petroleum ether and stirred for 30 min using a magnetic stirrer to remove fats and pigments. The residue was filtered using Vacuum Pressure Pump (Model No. 400-3912, Barnat company, Illinois, USA) and Whatman filter paper (Grade 4, 20-25 µm, Sigma-Aldrich, Australia). The residue was mixed with 10 mL/g of methanol and water solution (4:1, v/v) as a solvent using a magnetic stirrer for 30 min. The supernatant was drained and kept for 24 h at 4 °C. The precipitate from the supernatant was exposed to low-temperature drying (30 °C) for 48 h using an incubator (Premium blanket warmer, Thermoline Scientific, Wetherill Park, NSW, Australia).

Although 2% to 4% TE is commonly reported as effective for protein protection (Aerts et al 1999), a 6% level was also included to test the potential upper-limit response, as tannin effects vary with source and composition. The TE was mixed with a canola meal or soybean meal at 0%, 2% (20 mg/g), 4% (40 mg/g), and 6% (60 mg/g) DM for *in vitro* fermentation. Therefore, eight treatments were included, and a total of eight replicates of modules were used in two sequential runs for each treatment and parameters used. Six blank modules were also incubated with rumen fluid as background in each run. The TE was in powdered form; therefore, no carrier was required, and the 0% treatment consisted solely of the untreated meal.

### Rumen fluid collection and use

Two liters of rumen fluid was collected per run from four mid-lactation Holstein Friesian cannulated dairy cattle at Agriculture Victoria (Ellinbank, Victoria) in the morning before feeding and transported using the procedure described by Tunkala et al (2022). Cows were grazing perennial ryegrass (*Lollium perenne* L.) pasture, and wheat and barley grain mix (6 kg DM per day per cow) was supplied in the milking parlor. The rumen fluid was transported using an incubator (Premium blanket warmer, Thermoline Scientific, Wetherill Park, NSW, Australia) foreset on 39 °C and filtered using two layers of cheesecloth after arrival to the *in vitro* fermentation laboratory.

### Gas production method

The gas production method of Raab et al (1983) and Karlsson et al (2009), was used with minor modifications to fit into the ANKOM gas production system as detailed by Tunkala et al (2023b). A total of 10 g/L of rapidly soluble carbohydrates (3.33 g of maltose, 3.33 g of starch, and 3.33 g of xylose) were added to filtered rumen fluid as described by Aghajanzadeh-Golshani et al (2015) to minimise the background ammonia-N and stimulates microbial activity for 3 h in a 39 °C water bath (20 L Analogue Water bath, WB20; Ratek Instruments Pty Ltd, Boronia, VIC, Australia). Sodium bicarbonate (3.1 g dissolved in 63 mL of McDougall’s buffer per L of rumen fluid) was also added to the rumen fluid under continuous flushing of CO_2_.

The pre-incubated rumen fluid was mixed with McDougall (1948) buffer to obtain a buffered rumen fluid with a 1:2 rumen fluid to buffer ratio. A 500 mg DM of each feed was weighed into individual 250 mL ANKOM bottles, mixed with TE and 90 mL buffered rumen fluid, and incubated for 24 h in a 39 °C water bath. The gas pressure was recorded every 5 min by automated ANKOM gas production system and converted into gas volume using Avogadro’s law and ‘ideal’ gas law.

The *in vitro* degradable CP (IVDP, % total *N*) was calculated for each feed at 4, 8, 12, 16 and 24 h of gas production through intercepts of ammonia-N (*y*, mg/g) and gas production (*x*, mL/g), as described by Raab et al (1983) using the equation:


(1) $$\mathrm{IVDP}=\frac{\mathrm{AmmoniaN}\;\mathrm{at}\;\mathrm{zero}\;\mathrm{gas}\;\mathrm{production}(b^{0}\mathrm{intercept})-\mathrm{AmmoniaN}\;\mathrm{in}\;\mathrm{blank}}{\mathrm{Total}\;N\;\mathrm{of}\;\mathrm{incubated}\;\mathrm{feed}}\;$$

The proportion of protein fractions were estimated by fitting the IVDP to the nonlinear equations of Ørskov and McDonald (1979) using the exponential regression model of GenStat 21^st^ edition.


(2) $$Y\;=\;a\;+\;b\;\times \;\left(1\;-\;e^{-ct}\right)$$

Where: *Y* is the proportion of CP degraded at time *t*, fraction ‘*a*’ is the immediately available fraction of CP, fraction ‘*b*’ is the potentially degradable fraction of CP, and parameter ‘*c*’ is the degradation rate of fraction ‘*b*’. The undegraded protein fraction is calculated by subtracting the protein fraction ‘*a*’ and ‘*b*’ from the total CP as described by Tunkala et al (2023a).

### Analyses, measurements and sampling

The chemical composition of feeds were examined in a commercial laboratory (FeedTest Laboratory, Agrifood Technology, Werribee, Australia) using near infrared spectroscopy and the results were reported in Tunkala et al (2023c) for soybean meal and Tunkala et al (2024) for canola meal. The CP of the ­substrates was quantified by the Kjeldahl system. The post-fermentation pH value of the rumen fluid was measured using a pH meter (Oakton^®^ Acorn™ series pH 6 m, Sigma-Aldrich, North Ryde, Australia). Separate modules were used to collect 5 mL ammonia-N samples at 4, 8, 12, and 16 h of gas production with three replications and frozen at −20 °C until analysis. These modules were removed from the incubation after sampling. The modules from gas production samples were used as a source of ammonia-N samples at 24 h. Ammonia-N concentration was estimated by colourimetric technique as described by Weatherburn (1967) using a multiscan colourimetric plate reader (Thermo Multiskan Spectrum, Thermo Fisher Scientific, Australia).

Headspace gas samples were collected using the method described by Alvarez Hess et al (2019) for methane analysis by gas chromatography. At the end of each incubation run, separate gas samples were collected from the headspace of each bottle with an air-tight glass syringe (SGE International Pty Ltd, Ringwood, Vic, Australia), and transferred into a separate Exetainers^®^ (12 mL soda glass vial, Labco Ltd, Buckinghamshire, UK) which had previously been evacuated. Methane proportions in the headspace gas samples were determined by gas chromatography (GC). Headspace gas samples were analysed using Agilent 7890A equipped with 3 detectors (TCD, µECD, FID), and GILSON GX-271 auto sampler for transferring the pressurised sample from Labco Exetainers^®^ to the GC loops (1 mL × 2). The GC loops and sample inlet were flushed using helium between samples to avoid carryover. Columns used were: HayeSep^®^ N 80/100 mesh, 0.5 m × 1/8 in, SST (Precolumn for both channels); Porapak^®^ QS 80/100 mesh, 2 m × 1/8 in, SST (analytical on TCD—FID channel); HayeSep^®^ D, 80/100 mesh, 2 m × 1/8 in, SST (analytical to µECD).

A 5 mL liquid samples were also collected in three replicates per treatment after 24 h of incubation for volatile fatty acid (VFA) analysis using gas chromatography fitted with a flame ionisation detector with methyl valerate as the internal standard (Jouany 1982; Salas et al 2019).

The lag time (h) data was collected from the 24 h gas production spreadsheet for each ANKOM module. The gas production rate was computed using GenStat with the Gompertz model:


(3) $$Y\;=\;A\;+\;C^{\;\text{exp}\;\{}{}^{-\text{exp}[-B\left(X—M\right)]\}}$$

in which *A* is the *y*-intercept, *B* is the rate of gas production (mL/h), *C* is the maximum gas produced (maximum gas mL/g DM), *X* is the total time (h) of incubation, and *M* is the time (h) at which the maximum rate of gas production is reached.

### Statistical analysis

The mean differences of parameters between two substrates treated with four inclusion levels of TE from *Bauhinia hookeri* hay were analysed by two-factor ANOVA in GenStat 22^nd^ edition, considering runs as block. Each fermentation module was considered the experimental unit, and treatment means were compared across runs to ensure appropriate replication using the following model.


(4) $$Y_{ijk}=\;\mu \;+\;S_{i}+\;T_{j}+\;ST_{ij}+\;e_{ijk}$$

Where: *Y_ijk_* is the general mean of continuous dependent variables, *μ* is the mean value of treatments examined, and *S_i_* is the fixed effect of each substrate (*i* = soybean and canola meals) on the tested parameters, *T_j_* is the fixed effect of TE (0%, 2%, 4% and 6%) inclusion, ST_*ij*_ is the interaction effect between *S* and *T*, *e_ijk_* is the standard error term of the *k*^th^ observation from the (*i*, *j*)^th^ cell.

The data for ammonia-N and IVDP were analysed by three factor ANOVA, following the 2 × 4 × 5 factorial design from 2 feeds treated with 4 rates of TE and sampled at 5 different times. The three factor ANOVA model used was:


(5) $$Y_{ijk}{}_{l}=\;\mu \;+\;S_{i}+\;T_{j}+\;H_{k}+\;STH_{ijk}+\;e_{ijkl}$$

Where: *Y_ijkl_*, *μ*, *S_i_*, *T_j_* and *e_ijkl_* were described above, *H_k_* is the fixed effect of sampling time, STH_*ijk*_ is the interaction effect between *S*, *T* and *H*. Significant effects were declared at *P* < 0.05, and mean separation was performed when interaction was detected, using the least significant difference (LSD) test.

The linear regression and correlation coefficient between the TE inclusion in *X* axis and gas production and protein fraction ‘b’ in *Y* axis were determined using 2D scatter plot and Pearson’s correlation function of Genstat 22^nd^ Edition.

## Results

### In vitro fermentation characteristics

**Fig. 1.**
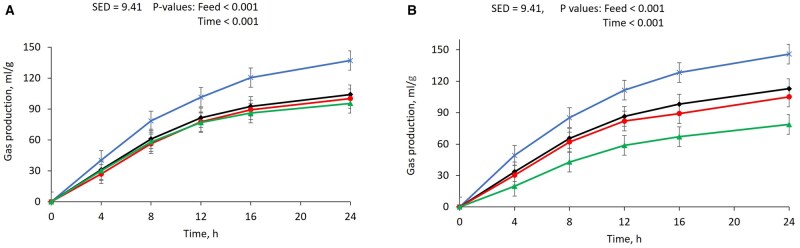
Gas production curves of feedstuffs a): soybean meal and b) canola meal treated with 0% (
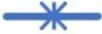
), 2% (
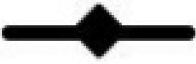
), 4% (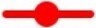) and 6% DM (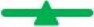) tannin extract from *Bauhinia hookeri* hay and incubated at 39 °C water bath using fresh rumen fluid. Data are least-square means of gas production after converting gas pressure into gas volumes using Avogadro’s law at varying incubation times (4, 8, 12, 16 and 24 h).

A decline in the volume and rate of gas production, pH value, and methane emissions were observed with increasing doses of TE for both feeds (Table 1 and Fig. 1). The gas production after 24 h of incubation was 141 mL/g DM for untreated soybean meal after 24 h of *in vitro* rumen fermentation, and the gas production of treated soybean meal was decreased by 24.2%, 29.5%, and 32.3% after mixing with 2%, 4%, and 6% TE, respectively, *P* < 0.001. Likewise, the untreated canola meal produced 146 mL/g DM gas after 24 h of incubation, and the gas production of treated canola meal was reduced by 16.4%, 30.1% and 52.7% in 2%, 4% and 6% TE inclusion when compared against the untreated treatment containing 0% TE. However, treatments did not influence gas production values for meals except for the 6% TE in canola meal. The gas production rate was greater for untreated soybean meal (5.85 mL/h) and canola meal (6.08 mL/h) than treated feeds (Table 1), *P* < 0.001.

**Table 1.** Effect of treating soybean and canola meals with 2%, 4% and 6% tannin extract from *Bauhinia hookeri* hay on *in vitro* fermentation parameters.

Substrates	Soybean meal				Canola meal					*P*-value^2^		
Rates of tannin (%)	0	2	4	6	0	2	4	6	SED^1^	S	T	S*T
**Cumulative gas production (mL/g DM)**	141	107	99.4	95.5	146	122	102	69.0	20.88	NS	*	NS
**Lag time (h)**	0.10	0.18	0.20	0.60	0.09	0.18	0.25	0.33	0.081	NS	*	NS
**Gas production rate (mL/h)**	5.85	4.45	4.14	3.98	6.08	5.07	4.26	2.88	0.978	NS	**	NS
**Methane (% gas)**	8.89	8.47	7.90	7.80	11.4	8.34	8.34	7.54	0.621	NS	*	NS
**CO_2_ (% gas)**	91.1	90.5	92.1	92.2	88.6	91.7	91.7	92.5	1.94	NS	*	NS
**pH**	6.82	6.80	6.75	6.73	6.78	6.77	6.74	6.72	0.021	NS	**	NS
**Total VFA (mM/L)**	86.6^c^	85.0^cd^	84.6^cd^	78.3^d^	111^a^	100^b^	88.0^c^	79.4^d^	6.71	***	***	***
**Acetic acid (mM/L)**	38.8^g^	41.8^d^	40.6^e^	31.4^h^	65.6^a^	57.0^b^	47.0^c^	39.4^f^	0.49	***	***	***
**Propionic acid (mM/L)**	7.82^f^	9.06^d^	9.0^d^	6.36^g^	11.9^a^	11.0^b^	9.32^c^	8.20^e^	0.145	***	***	***
**Butyric acid (mM/L)**	7.43^e^	7.52^e^	7.08^f^	5.87^g^	14.5^a^	11.8^b^	9.55^c^	7.69^d^	0.141	***	***	***
**Isobutyric acid (mM/L)**	1.29^e^	1.31^e^	1.25^f^	1.11^g^	2.14^a^	1.85^b^	1.58^c^	1.35^d^	0.015	***	***	***
**Valeric acid, mM/L**	27.4^b^	21.2^c^	21.9^c^	30.4^a^	9.87^g^	12.4^f^	15.6^e^	19.0^d^	1.07	***	***	***
**Isovaleric acid, mM/L**	3.95^d^	3.85^e^	3.57^f^	3.20^g^	7.26^a^	6.08^b^	4.95^c^	3.83^e^	0.099	***	***	***
**A:P ratio**	4.96^d^	4.92^d^	4.85^e^	4.93^d^	5.51^a^	5.21^b^	5.04^c^	4.81^e^	0.095	***	***	***

1 Standard error of the difference for Substrate (S) × Tannin extract (T).

2 Significance of effects of Substrate (S) × Tannin extract (T) and interactions: * *P* < 0.05; ** *P* < 0.01; *** *P* < 0.001; A:P ratio: acetic to propionic acid ratio.

a-d indicate mean separation; values sharing the same letter are not significantly different at P < 0.05.

The lag time increased for both meals following TE inclusion compared to untreated meals, *P* < 0.05. The untreated meals had positive gas yield within the first 0.1 h, and lag time did not differ between the soybean meals treated with 0% and 2% (0.18 h) TE. The soybean meal treated with 4% and 6% TE showed positive gas yield after 0.20 h and 0.60 h of *in vitro* fermentation, respectively. The lag time of canola meal treated with 2% (0.18 h), 4% (0.25 h) and 6% TE (0.33 h) was greater than the lag time of untreated canola meal (0.09 h).

Release of methane from soybean meal decreased by 4.73%, 11.1% and 12.3%, with the inclusion of 2%, 4% and 6% TE, respectively, *P* < 0.05. However, no statistical difference in methane proportion was observed between untreated soybean meal and that treated with 2% TE. Moreover, the TE inclusion reduced the methane proportion in headspace gas from canola meal by 26.8%, 27.5% and 33.9% with the inclusion of 2%, 4% and 6% TE, respectively, *P* < 0.05. The application of TE increased the proportion of carbon dioxide in canola meal when compared to untreated counterpart, *P* < 0.05. However, treatments did not influence carbon dioxide concentration in soybean meal. The post-fermentation pH of rumen fluid was reduced in treated meals when compared to the untreated treatments, except for 2% TE in soybean meal, where no effect was observed.

The total VFA concentration from untreated canola meal (111 mM/L) was greater than that of treated canola meals, *P* < 0.001. The total VFA concentration from treated canola meal was reduced by 9.91% at 2%, 20.7% at 4% and 29.0% at 6% of TE inclusion. Moreover, the untreated soybean and canola meal produced a larger acetic to propionic acid ratio (A:P) than treated meals, *P* < 0.001. However, the total VFA concentration was not significantly different between untreated (86.6 mM/L) and treated soybean meals, except for the 6% TE treatment (78.3 mM/L). The amount of butyric acid reduced with increasing dose of TE for both meals, *P* < 0.001. However, there was no difference between the concentration of butyric acid from untreated soybean meal and treated with 2% TE. There was an interaction between substrates and TE doses for individual VFA’s and A:P ratio, *P* < 0.001.

### Protein degradation

**Fig. 2.**
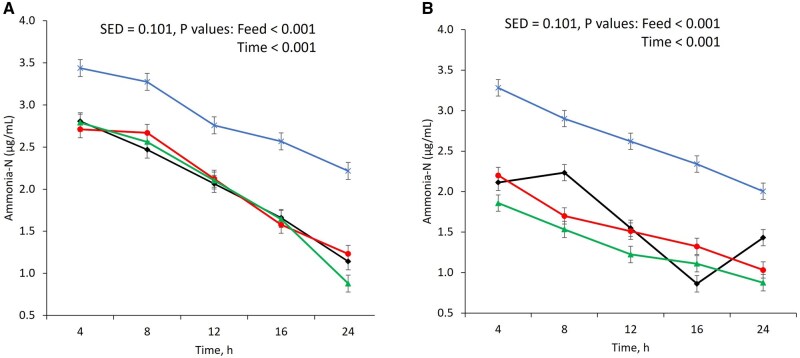
The amount of ammonia-N produced from a) soybean meal and b) canola meal treated with 0% (
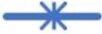
), 2% (
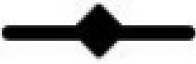
), 4% (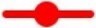) and 6% DM (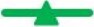) tannin extract from *Bauhinia hookeri* hay and incubated at 39 °C water bath using fresh rumen fluid. Data are least-square means of ammonia-N values measured at varying incubation times (4, 8, 12, 16 and 24 h).

The ammonia-N concentration was greater across all sampling periods for untreated meals and decreased with increasing duration of fermentation, *P* < 0.001, as shown in Fig. 2. The untreated soybean meal produced 3.44 µg ammonia-N per mL at 4 h and reduced to 2.22 µg ammonia-N per mL after 24 h. The ammonia-N produced from the untreated canola meal was 3.28, 2.90, 2.62, 2.34 and 2.01 µg ammonia-N per mL at 4 h, 8 h, 12 h, 16 h and 24 h sampling period, respectively, *P* < 0.001. Likewise, the ammonia-N concentration from treated soybean meal decreased with the time of fermentation. Treatments did not influence ammonia-N values in soybean meal. The ammonia-N values from canola meal treated with 6% TE (0.88 µg/mL) was lower than canola meal treated with 2% (1.43 µg/mL) and 4% (1.03 µg/mL) TE after 24 h *in vitro* rumen fermentation.

The IVDP values of untreated soybean and canola meals were greater than meals treated with 2%, 4% and 6% TE across fermentation periods, *P* < 0.001 (Table 2). There was no difference between IVDP of soybean meal treated with 2% (63%) and 4% (61%) TE after 24 h *in vitro* rumen fermentation; however, the 2% TE in canola meal produced a greater IVDP than 4%, *P* < 0.001. The IVDP was reduced from 6% tannin inclusion for both meals compared to other inclusion levels, *P* < 0.001. A substrate by time interaction was detected in the IVDP analysis as the substrates responded differently when 2% of TE was added to soybean and canola meals.

**Table 2.** The *in vitro* degradable crude protein (IVDP) of soybean and canola meals treated with 2%, 4% and 6% tannin extract from *Bauhinia hookeri* calculated using the intercept of gas production and ammonia-N values at 4, 8, 12, 16 and 24 h of *in vitro* fermentation.

Substrates	Soybean meal				Canola meal				SED^1^	*p*-value^2^			
Rates of tannin (%) DM	0	2	4	6	0	2	4	6		S	T	H	S*T*H
**Time (h) 4**	0.28	0.15	0.14	0.11	0.34	0.13	0.13	0.14	0.018	*	*	*	NS
**8**	0.35	0.19	0.19	0.17	0.41	0.21	0.19	00	…	…	…	…	…
**12**	0.58	0.34	0.32	0.28	0.53	0.22	0.21	0.27	…	…	…	…	…
**16**	0.76	0.49	0.46	0.31	0.74	0.33	0.23	0.28	…	…	…	…	…
**24**	0.92	0.63	0.61	0.54	0.87	0.52	0.48	0.45	…	…	…	…	…

1 Standard error of the difference for Substrate (S) × Tannin extract (T) × Time (H).

2 Significance of effects of Substrate (S) × Tannin extract (T) × Time (H) and interactions: * *P* < 0.05; the *P*-values in the first row are valid for the entire table.

The protein degradability parameters are presented in Table 3. The protein fraction ‘a’ of untreated soybean (23.8% CP) was larger than those of soybean meal treated with TE, *P* < 0.001. No difference was observed in protein fraction ‘a’ between soybean meals treated with 4% and 6% TE. The protein fraction ‘b’ was 61.2% CP for untreated soybean meal and increased with the 2% (66.3% CP), 4% (70.2% CP) and 6% (70.1% CP) tannin inclusion. The degradation rate for fraction ‘b’ of soybean meal was reduced from 9.55%/h to 4.76%, 2.93% and 2.92%/h after 0%, 2%, 4% and 6% TE inclusions, respectively, *P* < 0.01. The undegraded fraction remained unchanged between treatments.

**Table 3.** Protein fractions (a, b, undegraded) and degradation rate of fraction ‘b’ (c) of soybean and canola meals treated with 2%, 4% and 6% tannin extracted from bauhinia hookeri after 24 h *in vitro* rumen fermentation.

Substrates	Soybean meal				Canola meal					*P*-value^1^		
Rates of tannin (% DM)	0	2	4	6	0	2	4	6	SED^2^	S	T	S*T
Fractions and rate												
**a (% CP)**	23.8^d^	20.2^e^	15.1^f^	14.3^f^	58.2^a^	32.0^b^	28.8^c^	27.8^c^	2.53	***	***	***
**b (% CP)**	61.2^c^	66.3^ab^	70.2^a^	70.1^ab^	35.7	62.5^bc^	65.9^b^	66.1^ab^	4.18	***	***	***
**Undegraded (% CP)^3^**	15.0^ab^	13.5^b^	14.7^ab^	15.6^a^	6.10^c^	5.50^c^	5.30^c^	6.10^c^	2.121	***	***	NS
**c (%/h)^4^**	9.55	4.76	2.93	2.92	7.49	4.28	2.75	2.75	1.054	**	*	NS

1 Significance of effects of Substrate (S) × Tannin extract (T) and interactions:* *P* < 0.05; ** *P* < 0.01; *** *P* < 0.001.

2 Standard error of the difference for Substrate (S) × Tannin extract (T).

3 Calculated.

4 Degradation rate of protein fraction b.

a-d indicate mean separation; values sharing the same letter are not significantly different at P < 0.05.

The protein fraction ‘a’ of all treated canola meals were lower than untreated canola meal (58.2% CP), *P* < 0.001. There was no difference between protein fraction ‘a’ of canola meals treated with 4% (28.8% CP) and 6% (27.8%) TE. Similar to soybean meal, the protein fraction ‘b’ was greater in treated canola meals compared to untreated canola meal, *P* < 0.001. The canola meal treated with 0%, 2%, 4% and 6% TE showed 35.7%, 62.5%, 65.9%, and 66.1% CP fraction ‘b’ respectively. There was an interaction between substrates and TE doses for protein fraction ‘a’ and ‘b’ in both meals, *P* < 0.001. The degradation rate for protein fraction ‘b’ of untreated canola meal was 6.08%/h and reduced to 5.07%, 4.26% and 2.88%/h in 2%, 4% and 6% TE inclusion.

**Fig. 3.**
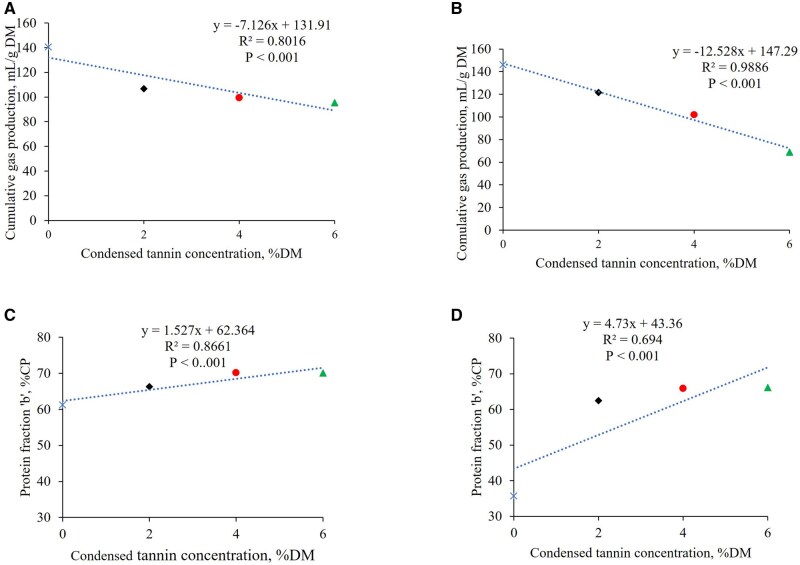
The linear regression equation and correlation coefficients (*R*^2^) between the condensed tannin concentration (% DM) in *X* axis and gas production (mL/g DM) from soybean meal a) and canola meal b), protein fraction ‘b’ (%CP) from soybean meal c) and canola meal d) in *Y* axis for 0% (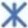), 2% (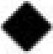), 4% (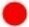) and 6% (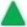) tannin inclusion per dry matter basis. The feeds were *in vitro* fermented using rumen fluid in 39 °C water bath for 24 h.

The increasing doses of TE showed linearly negative correlation with cumulative gas production (*R*^2^ = 0.80 in soybean meal and *R*^2^ = 0.99 in canola meal, *P* < 0.001, Fig. 3) and linearly positive correlation with protein fraction ‘b’ (*R*^2^ = 0.87 in soybean meal and *R*^2^ = 0.69 in canola meal, *P* < 0.001).

## Discussion

The major finding of this experiment was that *in vitro* fermentability of both substrates was reduced by the application of TE from *Bauhinia hookeri.* A reduction in the volume and rate of gas production, methane emission, ammonia-N concentration, and protein fraction ‘a’ indicates the potential *Bauhinia hookeri* as a TE source for methane reduction and CP protection against rumen degradation. The increase in lag time and protein fraction ‘b’ were also relevant indicators of protein protection activity during the *in vitro* fermentation process. The use of 4% TE showed more consistent results across fermentation and protein protection parameters among the inclusion levels tested.

The decline in volume and rate of gas production with increasing doses of TE, indicating reduced substrate fermentability and slower microbial activity in treated meals. This is consistent with Lavrenčič and Pirman (2021), who treated soybean meal with tannins extracted from chestnut wood at 60 g/kg of DM and found a reduction of gas production by 9%. Similarly, Tan et al (2011) found that increased levels of condensed tannin (20 to 60 mg/g) extracted from *Leucaena leucocephala* decreased *in vitro* gas production linearly by 22.8% to 46.2% for oven-dried guinea grass. Makkar et al (1995) demonstrated that the rate of *in vitro* fermentation was reduced by 4% in different hays using tannins extracted from *Acioa barteri*. The use of condensed tannin decreases total gas production by forming complexes from the bonding of tannin with feed molecules, which inhibit enzymatic and microbial activities (Getachew et al 2000; McSweeney et al 2001). However, the extensive reduction of gas production (52.7%) in canola meal from 6% TE inclusion could indicate potential detrimental effect at higher concentrations. The suppression of fermentation appears when higher load of tannins are supplied to rumen microorganisms beyond the tolerance limit (Kumar and Vaithiyanathan 1990; Makkar 2003).

Lag time (h) is the initial time during which the microorganisms colonise the substrates, multiply, and grow to enable the fermentation of the substrate and release gas (Ingraham et al 1983; Elghandour et al 2017). A prolonged lag phase indicates a delayed onset of microbial activity, postponing the initial degradation of complex substrates into simpler, more bioavailable forms (Tunkala et al 2023b). In this experiment, the lag time increased, and the onset of positive gas production was delayed with an increasing dose of TE. This finding agrees with the report of Salawu et al (1997), who fermented *Calliundra calothyrsus* leaf and stem *in vitro* with 50 g/kg of Quebracho tannins that resulted in a delay of gas production for 1.6 h in leaves and 2.1 h in stems. In addition, the use of 3%, 4.5% and 6% tannin extract from grape marc increased the fermentation lag time of soybean meal from 0.07 h to 0.09, 0.12 and 0.36 h, respectively (Alipour and Rouzbehan 2010). The extended lag time confirms that TE from *Bauhinia hookeri* has the potential to change the overall pattern of fermentation by slowing the activity of the microbial population responsible for gas production.

The reduction in methane emissions from meals treated with TE reflects the inhibitory effects of tannins on methanogenic activity. However, 2% TE was insufficient in soybean meal to impact methane percentage in the headspace gas. The study by Hassanat and Benchaar (2013) demonstrated that tannins extracted from chestnut, acacia or valonea at ≥50 g/kg DM resulted a reduction in methane production up to 40% from a total mixed ration composed of soybean meal in *in vitro* gas production system. Moreover, an *in vitro* study suggested the use of condensed tannin extracted from *Leucaena leucocephala* at 3% (15 mg/500 mg) combined with oven-dried guinea grass reduced methane production by 47% (Tan et al 2011). Tannins can modulate microbial composition through bactericidal effects and inhibit the activity of methane-producing microorganisms, such as methanogenic archaea, in the rumen fluid (Tavendale et al 2005). This finding implies that tannins from *Bauhinia hookeri* can be mixed with ruminant feed and used to reduce the environmental impact of livestock farming, as methane emissions from ruminants are a significant contributor to greenhouse gas emissions.

The total VFA concentration was not affected in soybean meal, except for 6% TE, and was significantly reduced in canola meal treated with TE. Makkar et al (1995) reported that Quebracho tannins decreased VFA production of hays from trees and shrubs fermented *in vitro* by 19.5% when added at 0.8 mg/mL, although no difference was observed for 0.4 mg/mL. On the other hand, the A:P ratio is often used as an indicator of rumen fermentation efficiency (Goel et al 2009) and was reduced significantly in treated canola meal. The changes in the VFA concentration and A:P ratio can be associated with shifts in the microbial population and the fermentation process caused by TE supplementation (Min et al 2020; Cai et al 2021). Moreover, the TE from *Bauhinia hookeri* can be used with soybean meal at 2% and 4% inclusion without affecting the energy supply for the ruminants. The difference in the impact of tannins on canola meal and soybean meal regarding total VFA concentration and A:P ratio showed that tannins might have distinct interactions with different nutrients. Differences in chemical composition and ability to react with phenolic bioactive compounds could be the reason for the interaction effect. Thus, the effect of TE in the feed fermentation could vary based on the substrate used, the source of proteins, amino acids composition, source and the dose of tannins (Makkar et al 1995; Grainger et al 2009; Krueger et al 2010).

The decrease in ammonia-N concentration with TE inclusion indicates reduced ruminal protein degradation due to tannin–protein complex formation. Śliwiński et al (2002) reported that application of 2.5 g/kg of chestnut tannins reduced ammonia by 21% in *in vitro* Rusitec fermentation of a basal diet containing soybean meal as a protein source. Moreover, Liu et al (2011) demonstrated that adding 3% (30 g/kg DM) of TE from a chestnut to a control diet with soybean meal reduced the ammonia-N concentration by 26.4% in sheep. Rumen microorganisms can readily utilise ammonia-N for microbial protein synthesis. However, high levels of ammonia-N can be detrimental to rumen function, animal health, and environmental pollution (Patra and Aschenbach 2018). The reduction in ammonia-N concentration indicates that tannins may have successfully decreased nitrogen availability for microbial use, resulting in lower ammonia production. Consequently, the review by Mueller‐Harvey (2006) concluded that an inverse relationship exist between the amount of ammonia and tannin content in the feed as a total tannin and polyphenol content have a harmful effect on ruminal microorganisms responsible for N metabolism (Pal et al 2015).

The shift in degradable and undegradable protein fractions in treated meals suggests enhanced protection of dietary protein from ruminal degradation. The use of tannins from *Cistus ladanifer* L. (15 and 30 g/kg DM) showed a decrease of fraction ‘a’ of soybean meal by 26.7% and an increase of fraction ‘b’ by 10.3% in *in situ* experiment using rams (Dentinho et al 2014). Despite the scarcity of information on *in vitro* studies about the effect of tannins in protein fractions ‘a’ and ‘b’, the report by Ramaiyulis et al (2019) indicated that use of tannin from *Uncaria gambir* leaf decreased rumen protein degradation by 16.8% in a mixed feed containing soybean meal fermented *in vitro*. Tannins promote the formation of protein-tannin complexes (Getachew et al 2000) or induce conformational changes in proteins and enzymes, leading to their decreased degradation (McSweeney et al 2001; Mueller‐Harvey 2006). This could result in a reduced availability of immediately available proteins for microbial degradation in the rumen fluid and an increased availability of potentially degradable proteins. Moreover, the potentially degradable proteins can be digested in the abomasum and small intestine, providing high biological value protein for high-producing or growing ruminants (Wang et al 1994, 1996). Therefore, the changes in protein fractions ‘a’ and ‘b’ can have positive implications for the nutritional value and utilisation of *Bauhinia hookeri* as a tannin source for protein protection.

The effectiveness of TE supplementation and the magnitude of changes in fermentation characteristics can depend on several factors. In the current experiment, the inclusion of 2% TE in canola meal reduced the 45% of protein fraction ‘a’ and increased 42.9% of fraction ‘b’ compared to the untreated canola meal. On the other side, the effect of 2% TE in soybean meal is lower than canola meal with 15.2% reduction of protein fraction ‘a’ and 7.7% increase in protein fraction ‘b’. This indicates that varying nutrient compositions and protein conformation between different feed ingredients can affect the interactions between tannins and the substrate (Patra and ­Saxena 2011) and could justify the interaction effect observed. Moreover, soybean and canola meals were evaluated as sole substrates in this *in vitro* experiment. Responses may differ in magnitude when protein supplements are included in mixed diets due to interactions with other dietary components. Future studies should therefore assess TE effects in total mixed rations to better reflect practical feeding conditions.

Increased concentrations of tannins used in the supplementation can negatively affect microbial population and feed degradation (Makkar et al 1995). Some tannins may also exhibit stronger inhibitory effects on microbial activity and binding effects on organic molecules, while others may have milder or more specific interactions (Grainger et al 2009; Patra and ­Saxena 2011). Furthermore, the composition of the microbial population in the rumen fluid can vary among animals and can be influenced by factors such as diet, genetics, season and management practices (Tajima et al 2001; Fernando et al 2010; Patra and Saxena 2011), which can result in different responses towards tannin supplementation, impacting the fermentation characteristics and the magnitude of changes observed. Therefore, considering the complexity of rumen fermentation and the interactions between tannins, substrates, and microbial populations, it’s crucial to account for these factors when applying tannin supplementation to ruminants.

## Conclusion

The *in vitro* rumen fermentation parameters in this experiment suggest the possibility of using TE from *Bauhinia hookeri* in canola and soybean meals; however, mixing the soybean meal with 2% TE did not alter methane proportion. The 6% TE inclusion was detrimental to simulated rumen fermentation, severely affecting gas production and VFA concentration in canola meal. Moreover, there was no difference between 4% and 6% TE in protein fractions of the meals. Therefore, 4% TE is the optimal concentration for application in *in vitro* rumen fermentation against excessive protein degradation in the rumen while achieving a safe methane reduction. However, *in vivo* experiments are required to evaluate the ideal *in vivo* inclusion rate.

## Abbreviations::

CP: crude protein
DM: dry matter
GC: gas chromatography
IVDP: in vitro degradable crude protein
TE: tannin extract
VFA: volatile fatty acid
